# RNA degradation in antiviral immunity and autoimmunity

**DOI:** 10.1016/j.it.2015.02.001

**Published:** 2015-03

**Authors:** Rachel E. Rigby, Jan Rehwinkel

**Affiliations:** Medical Research Council Human Immunology Unit, Medical Research Council Weatherall Institute of Molecular Medicine, Radcliffe Department of Medicine, University of Oxford, Oxford OX3 9DS, UK

**Keywords:** nonsense-mediated decay, RNA exosome, pattern-recognition receptor, type I interferon, Aicardi-Goutières syndrome, MDA5

## Abstract

Post-transcriptional control determines the fate of cellular RNA molecules. Nonsense-mediated decay (NMD) provides quality control of mRNA, targeting faulty cellular transcripts for degradation by multiple nucleases including the RNA exosome. Recent findings have revealed a role for NMD in targeting viral RNA molecules, thereby restricting virus infection. Interestingly, NMD is also linked to immune responses at another level: mutations affecting the NMD or RNA exosome machineries cause chronic activation of defence programmes, resulting in autoimmune phenotypes. Here we place these observations in the context of other links between innate antiviral immunity and type I interferon mediated disease and examine two models: one in which expression or function of pathogen sensors is perturbed and one wherein host-derived RNA molecules with a propensity to activate such sensors accumulate.

## Highlights

• The nonsense-mediated decay (NMD) pathway defends cells against RNA virus invasion.
• NMD targets viral RNAs for degradation, including by the RNA exosome.
• Genetic deficiencies in NMD and RNA exosome components cause autoimmunity.
• NMD and the RNA exosome prevent aberrant activation of innate immune responses.

## Post-transcriptional regulation of mRNA

The flow of genetic information from DNA to RNA to protein is a highly regulated process. This not only allows for gene products to be produced in appropriate amounts, at the right time points and in the correct locales, but also provides quality control. In many instances, post-transcriptional control of mRNA contributes to this regulation. In eukaryotic cells, post-transcriptional events can be divided into those that occur in the cell nucleus and those that take place in the cytoplasm. The former include splicing, capping, and polyadenylation of primary transcripts as well as nuclear export of processed mRNAs. Once in the cytoplasm, mRNAs may localise to specific areas within the cell, are translated and eventually degraded. mRNA translation and half-life vary greatly between transcripts and are controlled by a variety of mechanisms such as the miRNA pathway.

Other events in the cytoplasm, rather than controlling the amount of protein product produced from an mRNA, survey mRNA integrity and eliminate faulty transcripts. A well-studied example is the nonsense-mediated decay (NMD, see Glossary) pathway. Recent observations suggest that NMD not only surveys cellular transcripts but also recognises RNAs derived from viruses [1,2]. By targeting these transcripts for degradation, NMD counteracts virus infections and thus represents a new virus restriction pathway. We discuss these findings here in the context of the innate cell-autonomous immune response to virus infection. We also highlight potential implications of NMD and RNA decay in autoimmune-type diseases [3,4]. These include a role for NMD in increasing the activation threshold of innate signalling pathways by limiting the expression of pathogen sensors, as well as the possibility that nucleases such as the RNA exosome degrade cellular RNAs with a propensity to activate such sensors.

## Basic principles of NMD

**Figure 1:**
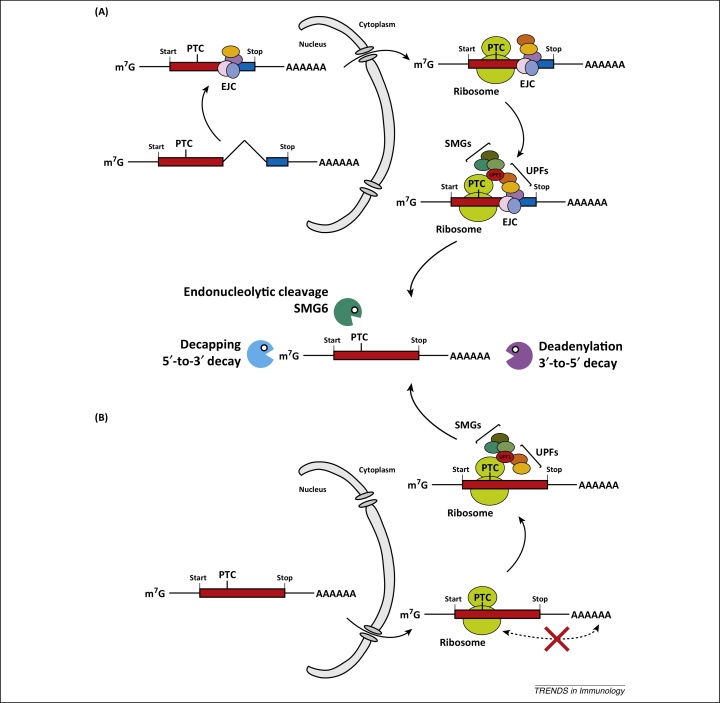
Nonsense-mediated decay (NMD). NMD recognises premature translation termination codons (PTCs) and this requires translation. The mechanism of PTC definition differs between species and individual transcripts. Two major pathways are shown here. (A) One important mechanism of PTC recognition relies on a protein complex called the exon-junction complex (EJC) [9]. EJCs are deposited on mRNAs during splicing and mark exon-exon junctions. EJCs are transported together with the mRNA into the cytoplasm and are removed from the mRNA during translation [82–84]. Importantly, most mRNAs contain the stop codon in their last exon; therefore, no EJCs are left on the mRNA when translation termination occurs. However, if mRNAs have a PTC upstream of the last exon, one or multiple EJCs remain on the mRNA at the moment when translation terminates. This constellation is recognised by a number of proteins including the essential NMD effectors UPF1, UPF2, and UPF3/3X. UPF1 (red) interacts with proteins involved in translation termination, while UPF2 (orange) and UPF3/3X (yellow) associate with the EJC. If an EJC is present downstream from a terminating ribosome, the UPF proteins interact to form a complex, SMG proteins (olive) are recruited, and degradation of the mRNA is initiated (middle panel) [5–8]. (B) EJCs are not always required for NMD [5–8]. Efficient translation termination requires interactions between proteins bound to the mRNA poly(A) tail and release factors, which associate with ribosomes at stop codons. If termination occurs at a PTC, the distance to the 3′-end and poly(A) tail may be too large to accommodate this interaction (red cross) [25,85–87]. This in turn is presumed to result in delayed release of the ribosome from the mRNA, allowing for assembly of UPF proteins and recruitment of SMGs independently of an EJC.

NMD is a highly conserved mRNA surveillance pathway and has been reviewed in detail elsewhere [5–8]. Here, we will discuss only the basic principles of NMD (Figure 1). NMD detects mRNAs harbouring premature translation termination codons (PTCs) and then targets these transcripts for degradation. PTCs can arise as a consequence of gene mutations or errors during transcription. If translated, PTC-containing mRNAs encode C-terminally truncated proteins. Such aberrant proteins can have adverse effects; for example, truncation may result in dominant-negative function. NMD therefore serves an important role in that it ensures that only intact mRNAs are translated.

How does NMD recognise faulty mRNAs bearing a PTC? NMD depends on protein translation and is triggered by ribosomes terminating translation in an ‘unusual’ position along the mRNA (Figure 1). Such unusual translation termination sites can be identified by different molecular mechanisms. One is based on the distance between a PTC and the poly(A) tail of an mRNA. Another mechanism involves a protein complex called the exon-junction complex [9]. Both mechanisms are further explained in Figure 1. Once a PTC has been identified, a number of proteins including the essential NMD effectors UPF1, UPF2, and UPF3/3X assemble to form a complex. Additional proteins involved in NMD then associate with the UPF proteins and initiate degradation of the mRNA. In animals, this can include the endonuclease SMG6, which cleaves the mRNA into two fragments, as well as SMG5 and SMG7, which recruit factors that remove the poly(A) tail and cap structure of the mRNA. As a result, free 5′- and 3′-ends are generated and this allows exonucleases to attack (Figure 1). One of these is the RNA exosome, a multiprotein complex that degrades RNA in a 3′-to-5′ direction [10,11].

## NMD restricts virus infection

Interestingly, NMD not only rids the cell of faulty, PTC-containing mRNAs but also regulates expression levels of a number of normal cellular transcripts. Indeed, around 10% of the transcriptome is regulated by NMD [12–18]. A number of features can predispose an mRNA to NMD, including long or intron-containing 3′ untranslated regions (UTRs), the presence of short open reading frames in the 5′ UTR or alternative splice events that introduce a PTC [19]. In these scenarios, translation terminates either upstream of an EJC or at a distance from the 3′-end, subjecting the transcript to NMD.

Many viruses produce RNAs with atypical features and this is often a result of their compact genome structures. For example, some viruses encode multiple proteins within one polycistronic mRNA molecule [20]. Translation of open reading frames at the 5′-end of such transcripts terminates far away from the 3′-end, a situation resembling a long 3′UTR. It is therefore conceivable that NMD detects viral RNAs [21] and – by targeting them for degradation – counteracts virus infection. This in turn is likely to drive evolution of viruses that escape or actively antagonise NMD.

A number of recent studies have validated these predictions. Screening for new virus restriction factors, the Helenius and Voinnet groups identified UPF1 in mammalian cells and plants, respectively [1,2]. In one of these studies, an siRNA screen revealed that depletion of UPF1 increases the susceptibility of human cell lines to infection with two positive-strand RNA viruses, Semliki Forest virus (SFV) and Sindbis virus [2]. Depletion of the NMD factors SMG5 and SMG7 also facilitates SFV infection [2]. Balistreri *et al*. further showed that silencing of UPF1 stabilises the SFV genomic RNA, although the molecular features that allow the NMD machinery to recognise viral RNA remain to be determined [2]. The other study employed a genetic screen in *Arabidopsis thaliana* and found that mutations in the gene encoding UPF1 facilitate infection with Potato virus X (PVX) [1]. Consistent with this observation, overexpression of a dominant-negative form of UPF1 increases susceptibility of *A. thaliana* to PVX infection and also enhances Turnip crinkle virus infection of *Nicotiana clevelandii* [1]. Viral genomic and subgenomic RNAs, which are both used as mRNAs, are more abundant in plant tissues as a result of impaired UPF1 function [1]. This effect is attributable, at least in part, to the presence of internal stop codons in the genomic RNAs of these two viruses and to long 3’UTRs in some of the subgenomic RNAs [1].

Taken together, these two studies using viruses with single-stranded, positive-sense RNA genomes show that NMD can detect and degrade some viral RNAs. NMD therefore constitutes a defence mechanism against infection with these viruses. It is interesting to ask if NMD might also have antiviral function against other classes of viruses. It is clear that the genomes of DNA viruses, retroviruses and negative-sense RNA viruses cannot be NMD targets because they are not translated. Indeed, Balistreri *et al.* found that depletion of UPF1 did not enhance infection of cells with respiratory syncytial virus or Uukuniemi virus, both of which have a negative-sense RNA genome [2]. Nevertheless, all viruses produce mRNAs and these may be recognised by NMD if they contain stop codons in unusual contexts. Consistent with this idea, certain retroviral transcripts are stabilised in cells with impaired UPF1 function [22–25]. Furthermore, some retroviruses appear to counteract NMD. One example is Rous Sarcoma virus: its unspliced RNA contains a stability element downstream of an internal stop codon [26]. Deletion of this RNA element predisposes the transcript to NMD [23,27]. It has been suggested that the Rous Sarcoma virus stability element interferes with UPF1 function, although the precise molecular mechanism remains to be determined [26]. Another virus interfering with NMD is human T-lymphotropic virus type 1, which deploys its Tax and Rex proteins to prevent NMD from degrading viral transcripts [22,24]. These observations show that some retroviruses antagonise NMD and as such are suggestive of an antiviral function of NMD. Whether mRNAs from DNA viruses are subject to NMD, and if NMD plays an *in vivo* role in limiting virus replication in mammalian hosts will be important to investigate. A recent study using hepatitis C virus (HCV) found that the viral core protein binds the EJC component WIBG (also known as PYM) [28]. This interaction displaces WIBG from the EJC and NMD efficiency appears to be reduced in HCV infected cells [28]. These observations might at first glance indicate that NMD restricts HCV and that the viral core protein counteracts this effect by targeting WIBG. Interestingly, however, WIBG depletion decreases HCV infection [28]. It may therefore be that WIBG is a cellular cofactor supporting the virus life cycle in a way unrelated to NMD and that NMD inhibition is simply a by-product of WIBG engagement by HCV.

It is tempting to speculate that other RNA quality control pathways could also contribute to cellular defence against virus invasion. Related to NMD are non-stop decay and no-go decay [29]. The former recognises mRNAs lacking a stop codon altogether, while the latter detects stalled ribosomes; both then target the transcript for degradation. It is possible that viral RNAs – perhaps due to secondary structure, codon usage or other unusual features – are predisposed to recognition by these decay pathways. Indeed, non-canonical translation mechanisms have been described for many different viruses [30]. In addition to NMD and other translation-dependent surveillance pathways, RNA decay and post-transcriptional control may be much more broadly implicated in controlling virus infection. Several viruses directly interfere with the expression or function of cellular RNases and other proteins involved in RNA degradation [21]. Moreover, RNAi (Box 1) is widely recognised as an ancient antiviral immune system operating in invertebrate animals and plants [31,32] and recent work suggests that virus control by RNAi may also contribute to host defence in mammals [33,34]. Post-transcriptional control pathways are therefore increasingly accepted to play important roles in controlling virus infection beyond their housekeeping functions in regulating cellular gene expression.

Another important question is how NMD integrates with other host defence responses. As discussed in further detail in the next section, cells are equipped to sense infection and to couple this to the induction of antiviral effectors. In mammals, many of these effectors are induced by type I interferon (IFN); this, however, does not appear to be the case for NMD factors, which are constitutively expressed and are not classified as interferon stimulated genes (ISGs) [35]. The antiviral function of NMD is therefore cell-intrinsic in that it does not require induction. NMD may thus represent a cellular barrier to virus infection that provides a first line of defence before virus sensing and induction of other effectors occur.

## Pathogen recognition, NMD, and autoimmunity

Across taxa, inducible cell-autonomous innate immune responses to infections are typically initiated by germline-encoded receptors often called pattern recognition receptors (PRRs). These receptors are activated by a variety of stimuli, including pathogen-associated molecular patterns (PAMPs), which were originally defined as conserved products of microbial biosynthetic pathways that are normally absent from host cells [36,37]. Some PRRs can also detect alterations to cellular homeostasis caused by infections [38] or are triggered by so-called danger-associated molecular patterns (DAMPs), endogenous molecules produced by or released from cells that die, become damaged or are stressed in the course of an infection [39]. Mammalian PRRs can be broadly divided into at least five groups [40,41]. Toll-like receptors (TLRs) and C-type lectin-like receptors (CLRs) are transmembrane proteins, located at the plasma membrane or in endosomes. In contrast, retinoic acid-inducible gene I (RIG-I)-like receptors (RLRs), cytosolic DNA receptors (CDRs), and NOD-like receptors (NLRs) are located in the cytosol. Receptors related to mammalian PRRs are expressed by many other organisms; for example, plants encode a variety of nucleotide-binding site leucine-rich repeat (NBS-LRR) proteins, which are related to mammalian NLRs.

In a study published back-to-back with those demonstrating an antiviral function of NMD [1,2], Gloggnitzer *et al*. investigated the role of NMD in the inducible innate immune response in plants [3]. An overview of pathogen recognition in plants is given in Box 2. A link between NMD and plant innate immunity had been suggested earlier: impairment of NMD results in severe phenotypes including seedling death, retarded growth and, interestingly, activation of immune responses [42–47]. These responses can be classified as ‘autoimmune’ given that they occur in the absence of infection. Building on an earlier study [47], Gloggnitzer *et al*. showed that the phenotype of SMG7-deficient *A. thaliana* can be rescued by introducing additional defects in the plant's NBS-LRR pathway [3]. These data demonstrate that NMD can prevent spontaneous and detrimental NBS-LRR responses in the absence of infection.

Theoretically, this observation can be explained in at least two ways. Firstly, NMD might downregulate the expression of NBS-LRRs or of proteins acting downstream of NBS-LRRs in this pathway. Absence of NMD would then result in a lower activation threshold and/or spontaneous signalling of the pathway, due to increased expression of its components. In support of this idea, altered cellular levels of PRRs can cause autoimmunity in mammals, as illustrated by duplication of the *Tlr7* gene in mice, which results in systemic autoimmunity modelling the clinical symptoms seen in the human autoimmune disease systemic lupus erythematosus (SLE) [48,49]. An alternative explanation is that NMD might interfere with signals that activate NBS-LRRs; for example, NMD may prevent the accumulation of ligands that trigger this pathway.

The first of these scenarios provides an explanation for autoimmunity in NMD-deficient plants. Gloggnitzer *et al*. showed that NMD plays a key role in downregulating mRNA levels of some NBS-LRRs belonging to the TNL subfamily and that this increases thresholds for activation of the plant effector-triggered immunity (ETI) response [3] (Box 2). Indeed, some TNL-encoding mRNAs have typical NMD-inducing features and display increased half-lives in NMD-deficient plants [3]. Utilising crosses between lines and plant genetics, Gloggnitzer *et al*. went on to show that RPS6, a TNL-subfamily NBS-LRR, can mediate autoimmunity in SMG7-deficient *A. thaliana* [3]. In the next section, we will compare these findings in plants with recent insights into a group of autoinflammatory and autoimmune diseases in humans called type I interferonopathies [50,110] and the links of these diseases with RNA biology.

## Type I IFN mediated diseases

**Figure 2:**
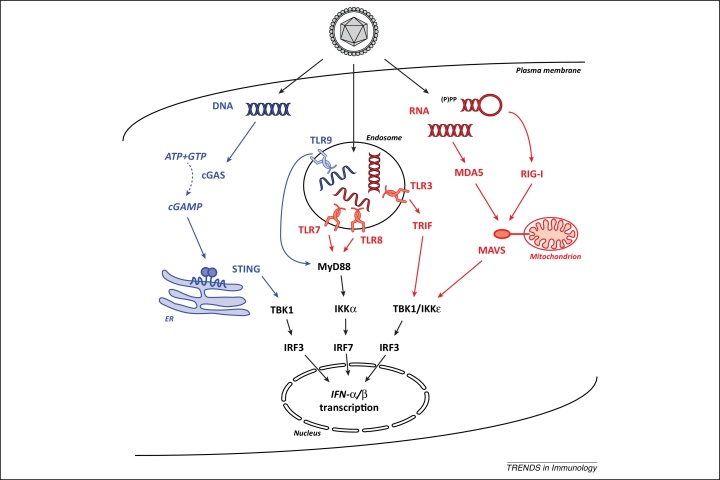
Nucleic acid detection by pattern recognition receptors. Virus infection delivers nucleic acids into infected cells. (Left) DNA is detected in the cytoplasm by cyclic GMP-AMP synthase (cGAS), which then uses ATP and GTP to generate cyclic GMP-AMP (cGAMP). This second messenger subsequently binds to the ER-associated protein STING which in turn activates TBK1. This kinase then phosphorylates the transcription factor IRF3, which forms a dimer and translocates to the cell nucleus to induce type I IFN gene transcription. Aside from cGAS, additional proteins including IFI16, DDX41, MRE11, and DNA-PK may participate in cytosolic DNA recognition [51] but are not shown here for simplicity. (Centre) Viral nucleic acids are also detected in endosomal compartments by TLR9 (DNA), TLR3 (dsRNA), and TLR7/8 (ssRNA). These PRRs signal through the adaptor proteins MyD88 or TRIF to activate the kinases IKKα, TBK1, and IKKɛ resulting in phosphorylation, dimerisation and translocation of IRF3 or IRF7. (Right) RNA in the cytoplasm of infected cells is recognised by RIG-I and MDA5, which interact with the mitochondrial protein MAVS to trigger TBK1/IKKɛ. Please note that a variety of other proteins are involved in signal transduction that are not shown here for clarity.

Nucleic acids are potent activators of the innate immune response in mammals and numerous specialised nucleic acid-sensing PRRs and their downstream signalling cascades have been identified [51] (Figure 2). The detection of viral and bacterial nucleic acids triggers a powerful innate immune response that is characterised by the production of type I interferons (IFNs). Type I IFNs signal *via* the type I interferon receptor to activate transcription of hundreds of ISGs, many of which restrict virus infection [35]. This thereby induces an antiviral state both in the infected cell as well as in the surrounding tissue microenvironment. The type I IFN-induced antiviral state prevents virus replication and spread and constitutes an important barrier to infection; this is evident, for example, from the fact almost every mammalian virus counteracts and/or evades the IFN response [52,53]. Moreover, type I IFNs facilitate cell-mediated innate and adaptive immune responses [54,55]. Type I IFNs are therefore crucial to successful immunity against virus infection.

However, a growing number of human autoinflammatory and autoimmune disorders are linked to type I IFNs, most notably SLE. Collectively termed type I interferonopathies [50,110], these pathologies are driven by chronic type I IFN production in the absence of virus infection (Table 1). Similar to what was discussed earlier for autoimmunity in plants, this could arise as a consequence either of perturbations in the pathways that induce type I IFN or of inappropriate generation and/or accumulation of nucleic acids able to activate PRRs [50,110].

## Aberrant STING and MDA5 activation

Examples for the first scenario are illustrated by mutations in *TMEM173* and *IFIH1*. Both of these cause inflammatory conditions characterised by elevated type I IFN levels (Table 1). The *TMEM173* gene encodes STING, a protein involved in the signal transduction cascade that leads to type I IFN induction in response to cytosolic DNA (Figure 2) [41]. Gain-of-function mutations in human *TMEM173* can give rise to a constitutively active STING protein that activates type I IFN in the absence of an upstream DNA trigger of this pathway [56,57]. *IFIH1* encodes MDA5, a PRR that responds to RNA agonists during virus infection (Figure 2) [51]. Heterozygous mutations conferring gain-of-function characteristics to MDA5 were identified in some patients with Aicardi-Goutières syndrome (AGS), causing type I IFN production in the absence of exogenous MDA5 stimulation [58,59]. This was attributed to enhanced binding of mutant MDA5 to RNA [58]. These data suggest that MDA5's activation threshold and/or specificity for RNA agonists are altered by AGS-associated mutations and imply the presence of an undefined endogenous RNA agonist. In keeping with this, a point mutation in *Ifih1* in mice results in spontaneous type I IFN-mediated lupus-like autoimmune disease in the absence of infection, although this appears to be caused by alterations in the conformation of MDA5 rather than an inappropriate response to an endogenous RNA ligand [60]. Nevertheless, this demonstrates further how dysregulated PRR signalling can lead to autoimmunity. This concept appears to be applicable across taxa, given that aberrant overexpression of some NBS-LRRs in plants (see above) results in autoimmune phenotypes [3].

## RNA editing controls IFN induction

In addition to *IFIH1* mutations, AGS can also occur as a result of mutations in any one of six other genes (*TREX1*, *RNASEH2A*, *RNASEH2B*, *RNASEH2C*, *SAMHD1*, and *ADAR1*) [111]. Chronic type I IFN production in all of these cases is likely to relate to the second scenario: the accumulation of aberrant nucleic acids with a propensity to activate PRRs. We will illustrate this with the recent example of ADAR1 and refer the reader to reviews for the other AGS-associated genes [61–63].

ADAR1 belongs to the protein family of adenosine deaminases acting on RNA (ADARs) that bind to double stranded RNA (dsRNA) and then convert adenosine to inosine [64]. This process is called RNA editing and can have a variety of biological outcomes; for example, inosine is decoded as guanosine during translation, resulting in amino acid substitutions that alter protein function [64]. The substrates of ADAR1 include both cellular and viral dsRNAs and ADAR1 has been reported to have both proviral and antiviral functions [64]. Two main isoforms exist in mammalian cells: p110 is constitutively expressed and p150 is IFN-inducible [65]. Mutations in human *ADAR1*, predicted to be hypomorphic, have been identified in patients with AGS and cause chronic IFN production [66]. Similarly, mouse embryos lacking Adar1 show clear evidence of a type I IFN response and die prenatally [67–69]. Some of the human mutations impair the RNA editing activity of ADAR1 to some extent *in vitro*, generally having a greater effect on the editing activity of the ADAR1-p150 isoform [66,70]. Although it remains unclear whether this is relevant to disease *in vivo*, it is tempting to speculate that non-edited RNAs accumulate in cells when ADAR1 function is impaired and that these RNAs in turn chronically activate PRRs. In line with this idea, cultured ADAR1-deficient cells show evidence of spontaneous type I IFN production and respond more strongly to RIG-I activation [71]. Conversely, ADAR1 overexpression in cells curtails RIG-I-dependent responses [71]. Consistent with this link between ADAR1 and RIG-I, *Adar1* knockout mouse embryos fail to induce type I IFN if they also lack Mavs, the signalling adaptor for RIG-I and MDA5 [70] (Figure 2). It is noteworthy that inosine containing RNA may also function as an antagonist of IFN induction [72].

## The RNA exosome and other nucleases prevent spontaneous IFN responses

Finally, we would like to highlight another situation in which accumulation of aberrant cellular RNAs results in chronic type I IFN production and disease. The RNA exosome is a multi-protein complex that degrades RNA in a 3′-to-5′ direction and plays a key role in RNA processing and surveillance pathways such as NMD [11]. An important activator of the RNA exosome in the cytoplasm is SKIV2L, an RNA helicase which forms the Ski complex together with two other subunits [11]. The Ski complex is thought to deliver RNA substrates to the catalytic core of the RNA exosome [11].

In studying a rare disease called trichohepatoenteric syndrome (THES), Eckard *et al*. found evidence that patients with loss-of-function mutations in the *SKIV2L* gene express elevated levels of type I IFNs [4]. This observation fits with a model in which SKIV2L-deficiency would result in the accumulation of RNA substrates normally degraded by the RNA exosome; these RNAs would then trigger activation of a nucleic acid sensing PRR (Figure 2). In line with this model, SKIV2L depletion confers an enhanced *in vitro* type I IFN response to exogenous RNAs known to activate RIG-I and MDA5 [4]. SKIV2L depletion in cells also results in type I IFN induction following experimental triggering of the unfolded protein response (UPR) [4]. The UPR naturally occurs when the levels of newly synthesised polypeptides in the ER exceed its protein-folding capacity [73]. Part of this stress response is the unconventional splicing in the cytoplasm of specific mRNAs encoding proteins involved in restoring homeostasis [74,75]. The RNA by-products of this splicing reaction had previously been shown to be capable of activating a RIG-I-dependent type I IFN response [76]. Consistent with these findings, Eckard *et al*. show that type I IFN induction in SKIV2L-depleted cells during ER stress is MAVS-dependent [4]. Taken together, these results suggest that SKIV2L acts as a negative regulator of the RNA-activated innate immune response by facilitating degradation of endogenous RNAs generated during the UPR. Failure of this degradation to occur can lead to unwanted type I IFN production and disease.

Interestingly, polymorphisms in *SKIV2L* have previously been linked with susceptibility to SLE [77]. This suggests that degradation of cellular RNAs by the RNA exosome may play a role in the pathogenesis of multiple autoimmune disorders, drawing parallels with TREX1, a 3′-to-5′ DNA exonuclease implicated in SLE and AGS [78,79]. TREX1-deficiency is likely to cause disease due a failure to degrade endogenous DNA species [61,80]. Once accumulated, these DNAs activate cytosolic DNA-sensing pathways and this results in type I IFN induction central to the aetiology of AGS and other interferonopathies [61,80]. The examples of SKIV2L and TREX1 therefore illustrate the importance of appropriate catabolism of potentially immunostimulatory self-nucleic acids to prevent autoimmunity.

## Concluding remarks

RNA decay pathways are increasingly recognised not only to control the quantity and quality of proteins produced by cells but also to contribute to host defence against infectious microorganisms, particularly viruses. We have illustrated this concept using the example of NMD. Indeed, in addition to its role in mRNA surveillance, NMD has recently been identified as a novel mechanism for cell-intrinsic virus control [1,2] and appears to function analogously to virus restriction factors. NMD also regulates the expression of factors involved in host defence such as PRRs [3]. It will therefore be important to study if NMD efficiency is regulated in the course of an infection (Box 3). Consistent with this idea, Gloggnitzer *et al*. found that bacterial infection or exposure to PAMPs reduces NMD efficiency and stabilises mRNAs targeted by NMD, including some TNL transcripts [3]. Similarly, Garcia *et al.* describe that endogenous NMD targets are stabilised in virus-infected plants [1]. It is interesting to ask if the host mediates inhibition of NMD during infection, allowing for increased expression of host proteins involved in the immune response, or if NMD is actively repressed by pathogens, avoiding degradation of the pathogen's own transcripts, as was shown for retroviral infections [22–25]. These two possibilities are not mutually exclusive, and the answer to this question will likely depend on the nature of the infecting microorganism. The complexity of the interactions between viruses and NMD is further underscored by the observation that the EJC component WIBG may be a cellular cofactor promoting HCV infection [28].

NMD deficiency in plants leads to an autoimmune phenotype attributable to increased PRR expression that results in aberrant induction of innate immune responses [3]. In humans, mutations affecting the RNA exosome – a nuclease that contributes to decay of NMD targets – result in chronic type I interferon production and disease [4]. This is likely to be due to PRR recognition of an endogenous RNA normally degraded by the RNA exosome. These two examples highlight different molecular causes for unwanted innate immune responses. In the former case, this is due to a lowered activation threshold and a consequently over-reactive and/or constitutively active innate signalling pathway, whilst in the latter case endogenous molecules with a propensity to activate PRRs build up in cells. These paradigms are relevant to a number of other human mutations that cause autoinflammatory and autoimmune diseases and we have illustrated this with the examples of *IFIH1* and *ADAR1*. Both of these genes are linked to AGS and encode proteins that either recognise and/or modify RNA. It is therefore becoming increasingly evident that the aetiology of AGS, and perhaps other diseases associated with aberrant type I IFN responses is, at least in some cases, intimately linked to RNA biology and RNA sensing pathways.

**Glossary**

**Aicardi-Goutières syndrome (AGS):** a genetically heterogeneous autoimmune disease predominantly affecting the brain and skin, which is characterised by the inappropriate activation of a type I IFN-mediated immune response, and which closely mimics congenital viral infection.
**Autoimmunity:** aberrant immune responses of an organism against its own cells and tissues. In the context of human disease, the term is typically reserved for conditions linked to adaptive B and T cell responses [81]. However, for the purpose of this article that covers different species from plants to humans, we use autoimmunity more broadly to include pathologies caused by both innate and adaptive immune responses.
**Effector-triggered immunity (ETI):** a plant immune response mediated by intracellular receptors, which is activated by the detection of pathogen effector molecules.
**Nonsense mediated decay (NMD):** a highly conserved mRNA surveillance pathway, which targets aberrant transcripts for degradation.
**Non-stop and no-go decay:** surveillance pathways that identify and degrade mRNAs, which lack a stop codon or are bound by stalled ribosomes, respectively. Thus far, these pathways have been primarily characterised in yeast.
**Pathogen-associated molecular patterns (PAMPs):** conserved components of microorganisms that are not normally found in host cells.
**Pattern recognition receptors (PRRs):** germline-encoded receptors of the innate immune system, which recognise PAMPs to trigger immune responses.
**PAMP-triggered immunity (PTI):** the first line of defence against pathogens used by plants, triggered by the recognition of PAMPs by PRRs present on the cell surface.
**RNA editing:** a process by which the nucleotide sequence of an RNA molecule is changed after its transcription, for example by deamination of adenosine to inosine.
**RNA exosome:** a protein complex that degrades RNA in 3′-to-5′ direction and consists of a barrel-shaped catalytic core and accessory proteins that recruit RNA substrates.
**Systemic lupus erythematosus (SLE):** a multisystem autoimmune disorder characterised by the presence of autoantibodies against nucleic acids and type I IFN production.
**Type I interferons (IFNs):** a family of secreted proteins (including IFN-β and multiple subtypes of IFN-α) that function as cytokines and signal *via* the type I IFN receptor to induce the expression of hundreds of interferon-stimulated genes (ISGs), many of which encode antiviral proteins.

## Figures and Tables

**Table 1:** Molecular causes of selected type I interferonopathies.

Gene (protein)	Disease^a^	Effect of mutations	Cellular consequences	Refs
*TREX1*	AGS, SLE, FCL	Loss-of-function	Accumulation of DNA in the cytoplasm, possibly derived from endogenous retroelements [61]	[78,79,103]
*RNASEH2A, RNASEH2B, RNASEH2C* (RNase H2)	AGS	Loss-of-function	Accumulation of ribonucleotides in genomic DNA [104], possible accumulation of RNA:DNA hybrids	[105]
*SAMHD1*	AGS^b^	Loss-of-function	Increased cellular dNTP pools	[106]
*ADAR1*	AGS	Altered or loss-of-function	Possible accumulation of unedited RNA	[66]
*IFIH1* (MDA5)	AGS^c^	Gain-of-function	Constitutively active MDA5 signalling	[58,59]
*TMEM173* (STING)	SAVI^d^	Gain-of-function	Constitutively active STING	[56]
*ISG15*	IBGC	Loss-of-function	Loss of negative regulation of type I IFN signalling	[107]

a Abbreviations: AGS, Aicardi-Goutières syndrome; SLE, Systemic lupus erythematosus; FCL, Familial chilblain lupus; SAVI, STING-associated vasculopathy with onset in infancy; IBGC, Idiopathic basal ganglia calcification.

b Also identified to cause cerebral vasculopathy and early onset stroke [108].

c Also linked to Single-Merton syndrome [109].

d Also identified in a family presenting with multiple systemic autoimmune diseases [57].

## Box 1 Antiviral RNA interference

RNAi is a post-transcriptional pathway that targets RNA molecules for degradation and/or translational repression [88]. Target transcripts are identified by base complementarity with short non-coding RNAs such as siRNAs or miRNAs, and this results in the recruitment of an RNA-induced silencing complex (RISC) to the target RNA. A key RISC component is a member of the Argonaute protein family. Some Argonaute proteins have endonuclease activity and directly cleave their target, while other Argonautes recruit additional factors to initiate RNA decay and/or translational repression.

The RNase III enzyme Dicer produces the short non-coding RNAs that guide the RISC to its targets. This is achieved by endonucleolytic cleavage of double-stranded RNAs (dsRNAs) or hairpin RNAs. These Dicer substrates are either encoded in the cell's genome, as is the case for miRNA precursors, or are exogenously delivered into cells. The latter scenario is particularly relevant to virus infection. Many viruses employ RNA-dependent RNA polymerases to replicate their genomes and this can results in the accumulation of long virus-derived dsRNA in cells [89].

Indeed, in invertebrate animals and plants, it is well established that RNAi constitutes a potent antiviral pathway [31,32]. Dicer recognises and cleaves viral dsRNA. This generates siRNAs, which are loaded into RISC that then targets viral transcripts, and this ultimately restricts the virus. It has recently been shown that at least some types of mammalian cells have the capacity to generate virus-specific siRNAs upon infection [33,34]. However, whether these siRNAs actually contribute to host defence in mammals and how antiviral RNAi and the type I IFN system relate to each other remain matters of debate [90–94].

## Box 2 Immunity in plants

The first line of defence against infection in plants is called PAMP-triggered immunity (PTI). PRRs present on the cell surface recognise conserved PAMP motifs and then activate signalling cascades, which culminate in the production of antimicrobial compounds [95,96]. These are sufficient to provide resistance against non-adapted pathogens; however, many pathogens introduce effector proteins into the cytoplasm of target cells to enhance infection by suppressing PTI and interfering with host signalling pathways. To counteract this, plants have evolved a second tier of pathogen detection termed effector-triggered immunity (ETI), which is deployed following infection by virulent pathogens. Intracellular ETI receptors, encoded by *Resistance* (*R*) genes, recognise pathogenic effector proteins either directly or *via* their activities within the cell [97]. This triggers signalling cascades and transcriptional reprogramming overlapping with those induced by PTI but with the added advantage of a rapid but sustained and robust response [98]. Most ETI receptors are members of a family of nucleotide-binding site leucine-rich repeat (NBS-LRR) proteins which share domains with mammalian NOD-like receptors (NLRs) [99,100]. Plant NBS-LRRs can be subdivided into two subfamilies: one with an N-terminal Toll/interleukin-1 receptor (TIR) domain, which shows homology to *Drosophila* Toll and human interleukin 1 receptor signalling domains (the TNLs), and one with a coiled-coil (CC) domain (CNLs) [101]. These two families utilise distinct signalling pathways to drive immune responses [102], which can include an oxidative burst, hormonal changes, upregulation of immune genes and a type of rapid cell death implemented to restrict pathogen growth, termed the hypersensitive response [98].

## Box 3 Outstanding Questions

• Does NMD defend cells against infection with DNA viruses, retroviruses or negative-strand RNA viruses? What is the contribution of NMD to antiviral immunity relative to other host defence pathways such as the type I IFN system, particularly in *in vivo* settings?
• Is NMD efficiency modulated by the infected host or by pathogens, and what are the outcomes of this regulation? Can NMD be targeted pharmacologically to boost innate immune responses?
• Aside from NMD and the RNA exosome, do other RNA quality control pathways (for example, non-stop decay and no-go decay) or other nucleases (for example, the 5′-to-3′ RNase XRN1) control viral infection or prevent accumulation of type I IFN-stimulatory nucleic acids?
• What is the origin of endogenous nucleic acids that trigger autoimmune and autoinflammatory diseases?
• What is the role of RNA quality control and RNA decay in controlling endogenous retroviruses and retrotransposons?
